# Supplementary material for: The interactome of intact mitochondria by cross-linking mass spectrometry provides evidence for coexisting respiratory supercomplexes
Source: Mol Cell Proteomics. 2017 Dec 8;17(2):216–32. doi: 10.1074/mcp.RA117.000470 (PMC5795388; doi:10.1074/mcp.RA117.000470)
Supplement: Supplemental Data [file supp_RA117.000470_133922_0_supp_23978_fzffwf.zip › spectra_annotation/mito_DR_spectra_annotation/495-1-14-1-22-1.pdf]

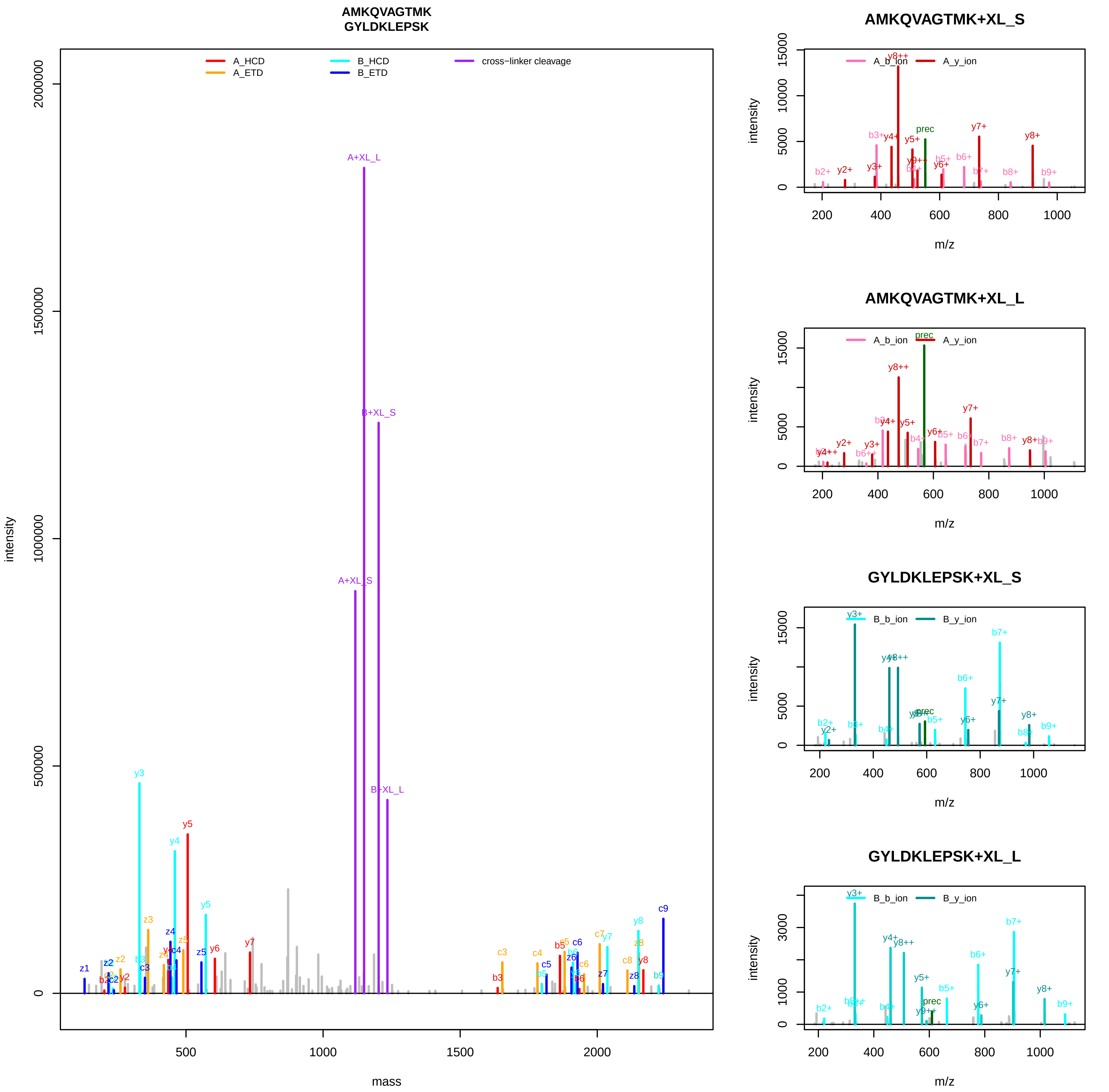
AMKQVAGTMK
GYLDKLEPSK
A_HCD
A_ETD
B_HCD
B_ETD
cross−linker cleavage
intensity
mass
0
500000
1000000
1500000
2000000
500
1000
1500
2000
A+XL_L
B+XL_S
A+XL_S
B+XL_L
y3
y4
y5
y6
y7
y8
z1
z2
z3
z4
z5
z7
z8
c2
c3
c4
c5
c6
c7
c8
c9
b3
b5
b6
b9
AMKQVAGTMK+XL_S
AMKQVAGTMK+XL_L
GYLDKLEPSK+XL_S
GYLDKLEPSK+XL_L
A_b_ion
A_y_ion
B_b_ion
B_y_ion
prec
m/z
200
400
600
800
1000
0
1000
3000
5000
10000
15000
y8++
y7+
y8+
b2+
y2+
y3+
b3+
y4+
y5+
b5+
b6+
b7+
b8+
b9+
y6+
y4++
y7+
b7+
y3+
y4+
